# Ponatinib promotes a G_1_ cell-cycle arrest of merlin/NF2-deficient human schwann cells

**DOI:** 10.18632/oncotarget.15912

**Published:** 2017-03-06

**Authors:** Alejandra M Petrilli, Jeanine Garcia, Marga Bott, Stephani Klingeman Plati, Christine T Dinh, Olena R Bracho, Denise Yan, Bing Zou, Rahul Mittal, Fred F Telischi, Xue-Zhong Liu, Long-Sheng Chang, D Welling Bradley, Alicja J Copik, Cristina Fernández-Valle

**Affiliations:** ^1^ Burnett School of Biomedical Sciences, College of Medicine, University of Central Florida, Lake Nona-Orlando, FL 32827, USA; ^2^ University of Miami Miller School of Medicine, Department of Otolaryngology, Miami, FL 33136, USA; ^3^ Center for Childhood Cancer and Blood Diseases, The Research Institute at Nationwide Children's Hospital and Department of Pediatrics, The Ohio State University College of Medicine, Columbus, OH 43205, USA; ^4^ Current Affiliation: Department of Otolaryngology, Massachusetts Eye and Ear Infirmary, Massachusetts General Hospital and Harvard University, Boston, MA 02114, USA

**Keywords:** neurofibromatosis type 2, schwannoma, PDGFR, SRC, STAT3

## Abstract

Neurofibromatosis type 2 (NF2) is a genetic syndrome that predisposes individuals to multiple benign tumors of the central and peripheral nervous systems, including vestibular schwannomas. Currently, there are no FDA approved drug therapies for NF2. Loss of function of merlin encoded by the *NF2* tumor suppressor gene leads to activation of multiple mitogenic signaling cascades, including platelet-derived growth factor receptor (PDGFR) and SRC in Schwann cells. The goal of this study was to determine whether ponatinib, an FDA-approved ABL/SRC inhibitor, reduced proliferation and/or survival of merlin-deficient human Schwann cells (HSC). Merlin-deficient HSC had higher levels of phosphorylated PDGFRα/β, and SRC than merlin-expressing HSC. A similar phosphorylation pattern was observed in phospho-protein arrays of human vestibular schwannoma samples compared to normal HSC. Ponatinib reduced merlin-deficient HSC viability in a dose-dependent manner by decreasing phosphorylation of PDGFRα/β, AKT, p70S6K, MEK1/2, ERK1/2 and STAT3. These changes were associated with decreased cyclin D1 and increased p27^Kip1^levels, leading to a G_1_ cell-cycle arrest as assessed by Western blotting and flow cytometry. Ponatinib did not modulate ABL, SRC, focal adhesion kinase (FAK), or paxillin phosphorylation levels. These results suggest that ponatinib is a potential therapeutic agent for NF2-associated schwannomas and warrants further *in vivo* investigation.

## INTRODUCTION

Neurofibromatosis type 2 (NF2) is a non-malignant tumor disorder affecting the peripheral and central nervous systems. Although bilateral vestibular schwannomas (VS) are a diagnostic hallmark of the disorder, NF2 patients typically develop multiple meningiomas, ependymomas and other schwannomas as well. VS lead to deafness, tinnitus, imbalance and can cause life-threatening brainstem compression [1]. NF2 is caused by mutations in the *NF2* gene that encodes the tumor suppressor protein known as merlin or schwannomin [2, 3]. Merlin belongs to the Band 4.1 family of proteins that link the actin cytoskeleton to membrane receptors and transporters. Merlin modulates the activity of multiple signaling pathways that control cell size, morphology, cell adhesion, proliferation, and survival. These include receptor tyrosine kinase (RTK; e.g. ErbB2/3, PDGFR, EGFR, HGFR), small GTPases, FAK/SRC, the mammalian target of rapamycin (mTOR)/PI3K/AKT, and Hippo pathways [4]. Currently, surgery and radiation are the mainstream treatment options for NF2-associated tumors. Depending on the tumor size and location, there are significant adverse effects associated with their removal. While an understanding of the biological functions of merlin is progressing, well-defined druggable molecular targets have yet to emerge. Increasingly, patients are treated off-label with the anti-angiogenic agent bevacizumab that also reduces edema in schwannomas without affecting the tumor cells. Dosing regimens are being optimized to reduce associated kidney toxicity observed with prolonged bevacizumab treatment [1, 5]. However, to date there are no FDA-approved therapies that target schwannoma cells directly and reduce morbidity and mortality of NF2 patients [1, 6].

Because of the slow-growing and benign nature of NF2 schwannomas, conventional chemotherapeutic agents are unsuccessful. Several RTK inhibitors have been investigated in preclinical studies and clinical trials with limited patient response. These include lapatinib (an EGFR/ErbB2 inhibitor; NCT00973739, NCT00863122), nilotinib (a PDGFR and c-kit inhibitor; NCT01201538), sorafenib (a VEGFR-2, PDFGRβ, and c-kit inhibitor), and axitinib (a VEGFR, c-kit, and PDGFRβ inhibitor; NCT02129647) [1, 7]. We selected ponatinib for evaluation because it is an FDA-approved drug that inhibits a relevant RTK, the PDGFR, and a downstream effector common to several *NF2* activated pathways, the non-receptor tyrosine kinase SRC. PDGFR and SRC signaling regulate cell survival, proliferation, migration and angiogenesis in many cell types [8, 9]. PDGFR is over-expressed and activated in VS and primary human schwannoma cells, consistent with merlin's role in downregulating surface levels of growth factor receptors [10–13]. In HEI-193 schwannoma cells, merlin overexpression inhibits cell proliferation by promoting PDGFR internalization and degradation [14]. There is evidence that SRC activity is deregulated in cells with loss of merlin function and thus is a candidate for therapeutic targeting. In human schwannoma cells, SRC activity is increased compared to normal Schwann cells, and in mouse glia cells, merlin inhibits proliferation by modulating SRC activity [15, 16]. Lastly, primary human schwannoma cells treated with the SRC inhibitor SU6656 exhibit decreased transcription of proliferation-associated genes [17]. Thus, an inhibitor that targets both PDGFR and SRC might have therapeutic value for NF2-associated tumors.

Ponatinib (AP24534, brand name: Iclusig^®^) is a third generation type IIA inhibitor of ABL/SRC tyrosine kinase (TK). It is orally active and initially received accelerated approval in 2012 for adult patients with chronic myeloid leukemia (CML) and Philadelphia chromosome-positive acute lymphoblastic leukemia (Ph+ ALL) that are T315I-positive and are not candidates for other TK inhibitors. Ponatinib binds the inactive, DFG-out (aspartic acid, phenylalanine and glycine) ABL/SRC conformation [18, 19]. In a cell-free kinase screen, ponatinib inhibited SRC with IC_50_ of 5.4nM and PDGFRα and PDGFRβ with IC_50_ of 1.1nM and 7.7nM, respectively [19].

In this study, we measured the ability of ponatinib to decrease proliferation and survival of merlin-deficient HSC and vestibular schwannoma cells with *NF2* mutations. We found that ponatinib caused a G_1_ cell-cycle arrest and mapped the regulatory signaling cascades modulated by the inhibitor. Our findings support further *in vivo* evaluation of ponatinib as a candidate drug for NF2 schwannomas.

## RESULTS

### Ponatinib decreases viability of merlin-deficient HSC and vestibular schwannoma (VS) cells

**Figure 1:**
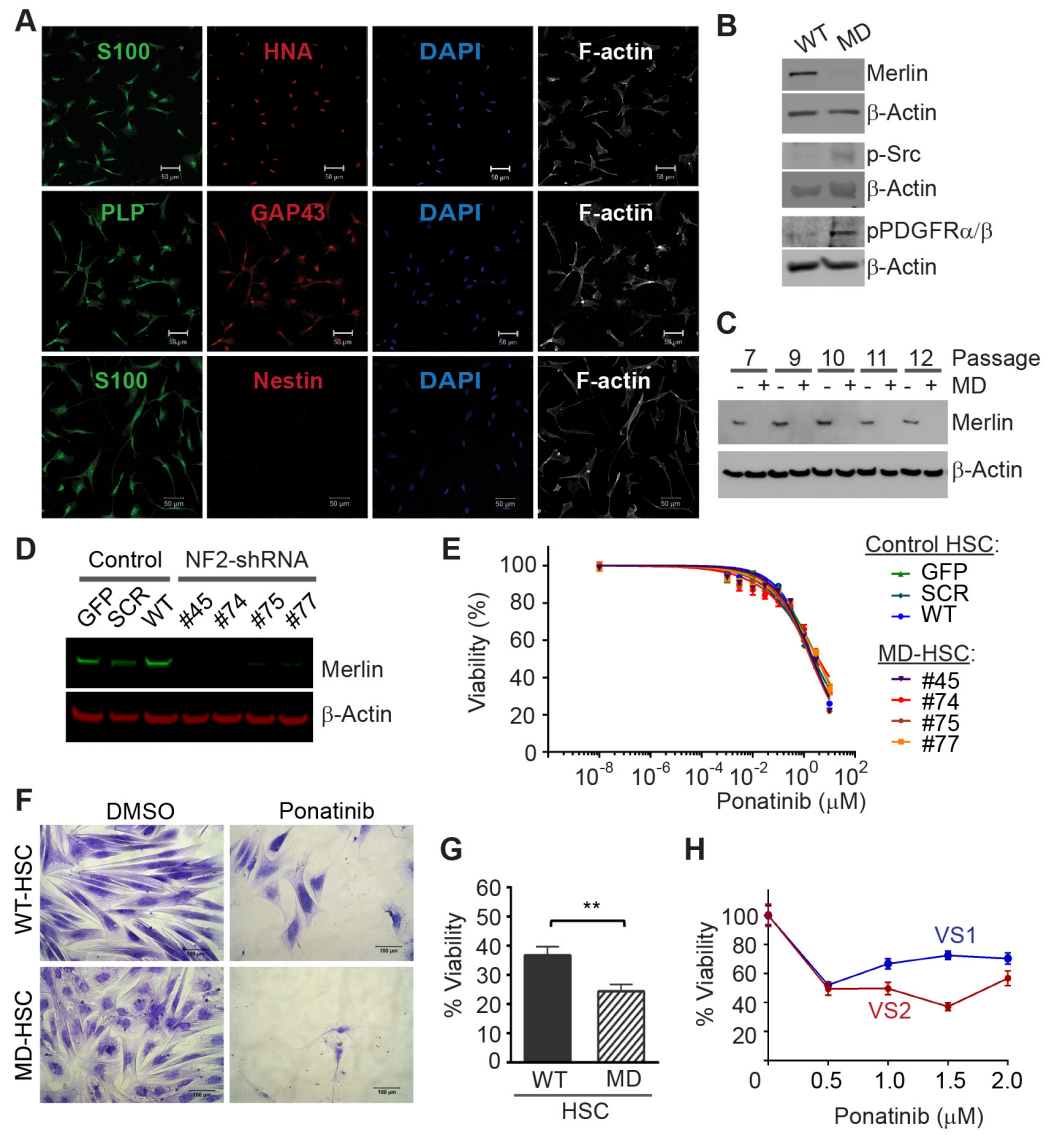
Ponatinib decreases HSC viability **(A)** Characterization of primary HSC. Confocal Images of HSC expressing human and SC linage markers: human nuclear antigen (HNA, red), S100 (green), GAP43 (red), proteolipid protein (PLP, green), negative nestin (red), DAPI stained nuclei (blue) and F-actin was visualized with phaloidin-Alexa633 (white). Scale bar: 50μm. **(B)** Representative Western blots of primary HSC and merlin-deficient HSC (MD-HSC) lysates, merlin silencing increased levels of phosphorylated SRC and PDGFRα/β. **(C)** Western blotting for merlin and β-actin in control HSC and merlin deficient cells at increasing cell passages. **(D)** Western blot for merlin and β-actin in three control HSC lines and four merlin-deficient (knock-down) HSC lines. **(E)** Ponatinib dose-response CellTiter-Fluor viability assay. Control HSC lines: SCR-HSC, GFP-HSC and WT-HSC and merlin-deficient HSC: #45, 74, 75, 77 were treated with increasing concentrations of ponatinib in constant 0.1% DMSO or vehicle alone for 48h. Viability is presented as a % of the DMSO control. Graph represents the mean ± SEM of three independent experiments. **(F-G)** WT-HSC and MD-HSC (#45) were maintained in serum free medium for 5-10 days and then treated with 0.25μM ponatinib for one week. Relative cell numbers was assessed using a crystal violet assay: **(F)** Representative 20X phase contrast images of cells. Scale bar= 100μm. **(G)** Cell viability calculated as a % of their respective DMSO control. Graph represent mean ± SEM of three independent experiments (** *p*<0.01, unpaired *t-*test, two tailed). **(H)** Viability of primary human VS cells treated for 48h with increasing ponatinib concentrations. Relative cell viability was assessed using a crystal violet assay and presented as % viability normalized to DMSO group. Plot of mean ± SEM of 6 replicates. VS1 (heterozygous deletion of 23 nucleotides in exon 8 of the *NF2* gene, non-irradiated, passage 2); VS2 (heterozygous missense c.1460T>A and p. I487N in exon 14 of the *NF2* gene, non-irradiated, passage 2).

To create a suitable cell line for drug discovery studies, we first authenticated primary HSC based on their expression of human nuclear antigen and Schwann cell markers, S100, PLP, and O4 (Figure 1A). We then used lentiviral delivery of NF2-shRNA to reduce expression of merlin in the primary HSC. Merlin levels were stably reduced to nearly undetectable levels in the transduced cells compared to the parental HSC (Figure 1B, C). The merlin-deficient HSC did not contact inhibit but did not form aggregates and grows in multiple layers; many of these merlin-deficient HSC maintained an elongated morphology when cultured in the presence of serum and mitogens (Supplementary Figure 1AB). We measured basal levels of PDGFRα/β and SRC phosphorylation in primary HSC prior to and following knockdown of merlin. We found that depletion of merlin expression was associated with increased levels of p-PDGFRα/β and p-SRC compared to the parental HSC (Figure 1B). This finding agrees with previous reports of increased activation of the PDGFR and SRC pathways in human schwannomas compared to normal human nerve [10, 20, 21].

We screened the ability of ponatinib to reduce viability of multiple control and merlin-deficient HSC lines. As controls, we tested the parental wild-type HSC (HSC-WT), HSC expressing a scrambled shRNA construct (HSC-SCR), HSC expressing a Turbo-GFP shRNA (HSC-GFP), and merlin-deficient HSC lines (MD-HSC) expressing shRNA sequences that target the human *NF2* gene, (MD-HSC #45, #74 #75 and #77). All control HSC expressed merlin, whereas the HSCs transduced with shRNA constructs targeting the *NF2* gene had nearly undetectable merlin levels (Figure 1D). We performed 48 hour dose-response viability assays on the seven HSC lines in complete growth medium containing serum and growth factors. The results indicated that ponatinib reduced viability of all of the cell lines in a dose-dependent manner (Figure 1E). Under the conditions tested, ponatinib was not selective for MD-HSC over scrambled, GFP or untransduced HSC (IC_50_ HSC-SCR= 3.3μM, HSC-GFP=2.4 μM, HSC-WT= 2.3 μM, MD-HSC#45= 2.2 μM, MD-HSC#74= 3.3 μM, MD-HSC#75= 1.9 μM and MD-HSC#77=3.7 μM). The average maximal response at 10μM was a 71% decrease in cell viability. However, when control merlin-expressing cell line (HSC-WT) and merlin-deficient HSC (MD-HSC#45) were cultured in the absence of serum and mitogens, merlin-deficient HSC were significantly more sensitive to 0.25 μM ponatinib than the merlin-expressing HSC (Figure 1F,G).

We tested ponatinib's effect on cultured human vestibular schwannoma cells with *NF2* mutations. We assessed relative cell viability using a crystal violet assay following 48 hour incubation with ponatinib. Cell viability was reduced by approximately 40% at 2μM in VS1 and VS2 compared to DMSO-treated cells in agreement with the IC_50_ obtained with our MD-HSC lines. (Figure 1H).

### Ponatinib decreases viability of merlin-deficient HSC independent of the SRC/FAK/paxillin pathway

**Figure 2:**
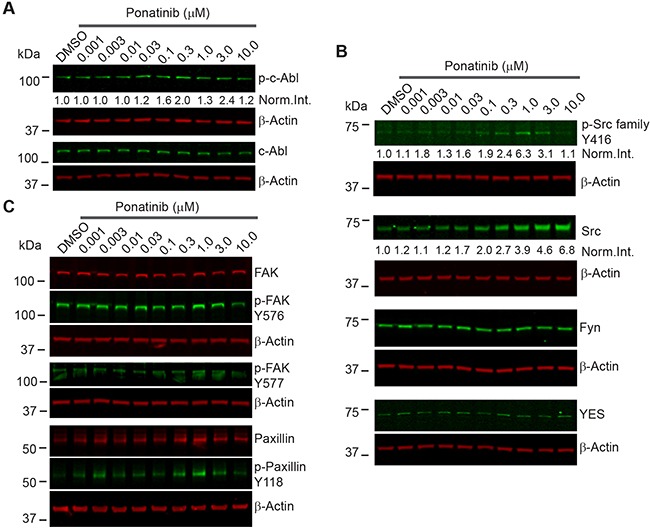
Ponatinib decreased merlin-deficient HSC viability independent of ABL/SRC/FAK pathway inhibition Representative ponatinib dose-response Western blots (n=3). MD- HSC#45 plated in 12-well plates were treated with increasing concentrations of ponatinib for 2h as indicated. Cells were harvested, lysed, resolved by SDS-PAGE and blotted for: **(A)** p-ABL-Tyr452, c-ABL and β-actin as a loading control; **(B)** p-SRC-Tyr416 and total SRC, FYN, YES, p-FAK-Tyr576, p-FAK-Tyr577, total FAK, p-Paxillin-Tyr118, and total paxillin. The β-actin levels were used as loading controls.

Ponatinib did not reduce net levels of ABL phos-phorylation in merlin-deficient HSC (Figure 2A). Ponatinib induced a dose-dependent increase in the total SRC protein level but did not alter the levels of the other SRC family members, FYN and YES, that play important roles in SC biology as well (Figure 2B) [22, 23]. We found a slight increase in SRC-Tyr416 phosphorylation in merlin-deficient HSC treated with 0.3 to 3μM (with a peak at 1μM). The phosphorylation pattern, however, did not coincide with the increase in the SRC protein levels (Figure 2B).

Key effectors transducing extracellular matrix adhesion and growth factor-dependent stimuli in Schwann cells are the SRC substrates, FAK and paxillin, a focal adhesion-associated adaptor [24]. FAK is a key mediator of extracellular matrix-integrin and RTK signaling that is upregulated in schwannomas [10]. Upon FAK autophosphorylation at Tyr397, SRC binds the phosphorylated residue and phosphorylates FAK on Tyr576 and Tyr577, resulting in stabilizing the activation loop of FAK in the active conformation and its binding to substrates, thereby providing FAK with maximal activity [25]. In turn, FAK either directly or via SRC, phosphorylates paxillin at Tyr118 [26]. We therefore assessed FAK and paxillin phosphorylation in merlin-deficient HSC treated for 2h with increasing ponatinib concentrations. Western blot analysis showed that ponatinib did not reduce FAK-Tyr576 or Tyr577 or paxillin-Tyr118 phosphorylation similar to the SRC-Tyr416 phosphorylation pattern (Figure 2C and Supplementary Figure 2). Together the results demonstrate that the ABL, SRC, FAK and paxillin pathways are not inhibited by ponatinib in merlin-deficient HSC.

### Ponatinib decreases activation of the PDGFRα/β, PI3K, MEK1/2, ERK1/2 and STAT3 signaling pathways

**Figure 3:**
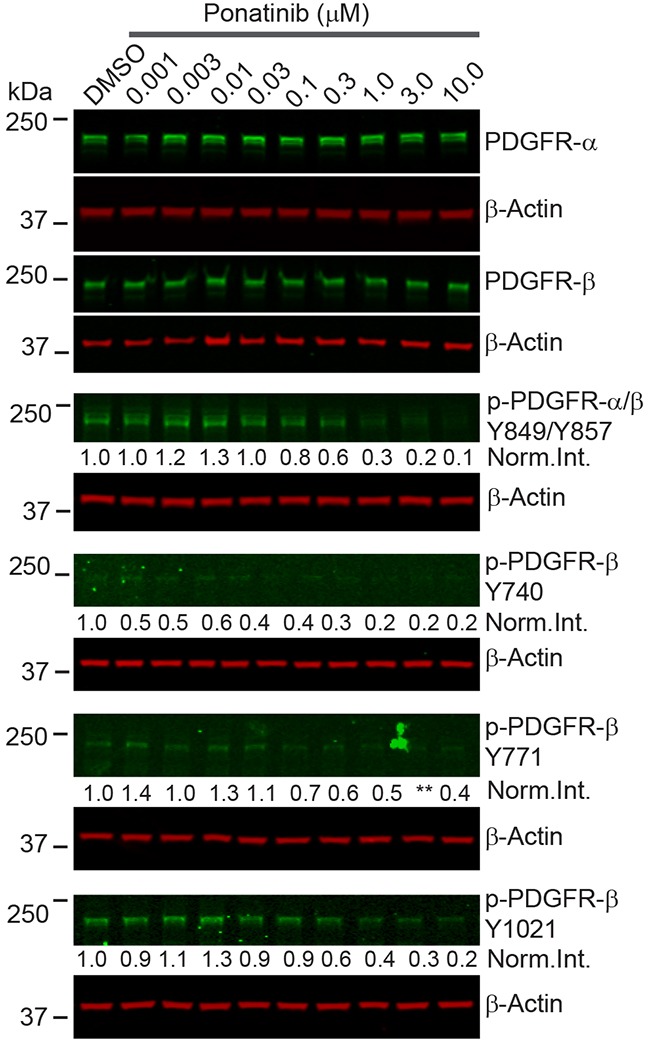
Ponatinib inhibited PDGFRα/β phosphorylation in merlin-deficient HSC Representative ponatinib dose-response Western blots (n=3). MD-HSC#45 plated in 12-well plates were treated with increasing concentrations of ponatinib for 2h as indicated. Cells were harvested, lysed, resolved by SDS-PAGE and blotted for p-PDGFRα/β-Tyr849/Tyr857, p-PDGFRβ-Tyr740, p-PDGFRβ-Tyr771, p-PDGFRβ-Tyr1021 and total PDGFRα and PDGFRβ. The β-actin levels were used as loading controls.

**Figure 4:**
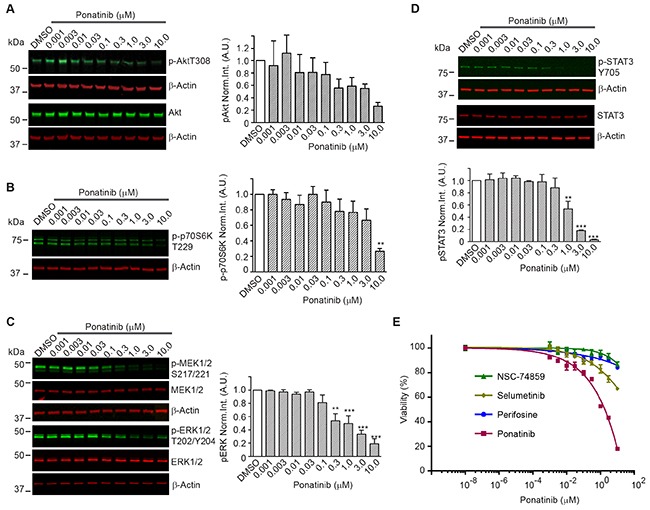
Downstream signaling pathways inhibited by ponatinib in merlin-deficient HSC Western blots of extracts prepared from merlin-deficient HSC#45 treated with increasing ponatinib concentrations. Quantitation was done by fluorescence intensity analysis, normalized to β-actin, and plotted as mean ± SEM (n=3). One-way analysis of variance and Dunnett's multiple comparison post-test were used for statistical analysis (* *p*<0.1; ** *p*<0.01 and *** *p*<0.001). Representative Western blots of dose response experiments at 2h for: **(A)** p-AKT-Thr308 and AKT; **(B)** p-p70S6K-Thr229; **(C)** MEK1/2, p-MEK1/2-Ser217/Ser221, ERK1/2, and p-ERK1/2-Thr202/Tyr204; **(D)** p-STAT3-Tyr705, and total STAT3. (**E)** Ponatinib dose-responseCellTiter-Fluor viability assay. MD- HSC#45 were treated with semi-log serial dilutions of ponatinib, NSC-74859, selumetinib, perifosine in 0.1% DMSO for 48h, or vehicle alone. Viability is presented as % of the DMSO control. Graph represents the mean ± SEM of three independent experiments.

To identify the signaling pathways modulated by ponatinib, we conducted a series of Western blots of merlin-deficient HSC treated for 2 hours with increasing concentrations of ponatinib. We found that ponatinib reduced phosphorylation of PDGFRα/β at Tyr849/Tyr857, the autophosphorylation sites in the activation loop of these kinases, in a dose-dependent manner without altering the PDGFRα/β protein levels (Figure 3). In addition, ponatinib reduced phosphorylation of three additional tyrosine residues in PDGFRβ at positions 740, 771 and 1021 (Figure 3). Phosphorylation of these residues increases affinity for binding and activating PI3K, SRC, the GTPase Activator of Ras (GAP), GRB2, and PLCγ [27]. When cells are stimulated with growth factors, AKT (also known as protein kinase B) and p70 S6 kinase are activated in a phosphatidylinositol 3-kinase (PI3K)-dependent pathway. Thr308 in the AKT activation loop and Thr229 in the p70S6 kinase catalytic domain are phosphorylated by 3-phosphoinositide-dependent protein kinase-1 (PDK1) *in vivo* and *in vitro* [28, 29]. Therefore to probe activity of the PI3K pathway in ponatinib-treated cells, we assessed AKT-Thr308 phosphorylation and p70S6 kinase-Thr229 phosphorylation by Western blots. We found that both AKT-Thr308 and p70S6 kinase-Thr229 underwent a dose-dependent decrease in phosphorylation (Figure 4A,B).

Previous studies demonstrated that overexpression and activation of PDGFRβ strongly activates MEK1/2 and ERK1/2 in human schwannoma cells leading to enhanced proliferation [10]. We assessed the phosphorylated MEK1/2 and ERK1/2 levels in merlin-deficient HSC after ponatinib treatment. We found that ponatinib treatment was associated with a dose-dependent decrease in MEK1/2-Ser217/Ser221 and ERK1/2-Thr202/Tyr204 phosphorylation in merlin-deficient HSC (Figure 4C).

Although net inhibition of SRC phosphorylation was not observed, ligand binding to PDGFR promotes receptor binding to SRC and recruitment of STAT3, followed by Tyr705 auto-phosphorylation of STAT3 and its dimerization and translocation into the nucleus to directly drive gene expression needed for cell proliferation [30–32]. Western blot analysis showed that ponatinib decreased STAT3 phosphorylation in a dose-dependent manner in merlin-deficient HSC (Figure 4D).

Lastly, to further evaluate the contribution of PI3K/AKT, MEK, or STAT3 inhibition downstream to PDGFR responsible for ponatinib's effect, we individually inhibited AKT with perifosine/KRX-0401, MEK with selumetinib/AZD6244, and STAT3 with S3I-201/NSC-74859 and compared the results to ponatinib's effects on cell viability. MEK, AKT or STAT3 inhibition alone only partially decreased merlin-deficient HSC viability. Inhibition of STAT3 with S3I-201 was the least effective, and although selumetinib was more efficacious than perifosine and S3I-201, none of individual inhibitors even at the highest concentration tested (10 μM) matched ponatinib's efficacy (Figure 4E). These results suggest that simultaneous inhibition of these three pathways occurs in response to ponatinib and similarly contributes to the loss of viability of merlin-deficient HSC.

### Ponatinib arrests merlin-deficient HSC in G_1_ by decreasing cyclin D1 and increasing p27^Kip1^ levels

**Figure 5:**
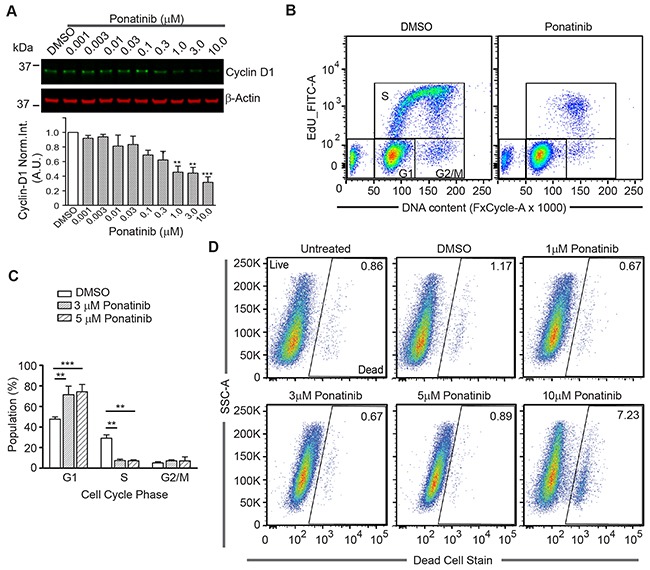
Ponatinib arrests merlin-deficient HSC at the G_1_ phase of the cell cycle **(A)** Representative Western blots for Cyclin D1of lysates prepared from merlin-deficient HSC#45 treated 24h with increasing concentrations of ponatinib. Plotted below as mean ± SEM (n=3). One-way analysis of variance and Dunnett's multiple comparison post-test were used for statistical analysis (** *p*<0.01 and *** *p*<0.001). **(B,C)** Merlin-deficient HSC were treated with 3 and 5μM ponatinib for 24h and during the last 3h, 10μM EdU was added. Cells were harvested, labeled with live/dead fixable dye, and analyzed by flow cytometry. **(B)** Representative plots of the distribution of EdU- and FxCycle-labelled cells of 0.1% DMSO vehicle control and ponatinib treated cells. **(C)** Graph of the distribution of the cell cycle phases (gated for the live population) of all the experiments as mean ± SEM, n=4; **p<0.01 and ***p<0.001 were determined by two-way ANOVA and Bonferroni multiple comparisons post-test. **(D)** Representative plots of the distribution of live and dead cell population in these experiments with increasing concentrations of ponatinib as indicated.

A molecular link between ERK1/2 and STAT3 to proliferation is through cyclin D1 to regulate G_1_-to-S cell cycle progression. ERK1/2 activity is required for expression of cyclin D1 in the G_1_ phase of the cell cycle. Moreover, STAT3 transcriptionally regulates cyclin-D1 by binding to its promoter region [33, 34]. We assessed the level of cyclin D1 in merlin-deficient HSC after a 24h incubation with ponatinib. We found that ponatinib strongly decreased cyclin D1 protein levels in a dose-dependent manner (Figure 5A). These results suggest that ponatinib reduces the viability of merlin-deficient HSC by inhibiting PDGFRα/β-dependent activation of MEK/ERK and STAT3 pathways, leading to decreased cyclin D1 expression.

To test the possibility that the decrease in the viability of merlin-deficient HSC by ponatinib was a consequence of cell-cycle blockage due to reduced cyclin D1 expression, we analyzed the distribution of cells among the different phases of the cell cycle. There was a significant increase in the number of cells in the G_1_ phase when treated with 3 and 5μM ponatinib compared to vehicle controls (72% ±8% and 74% ±7% vs. 47% ±2% of control). This was accompanied by a concomitant decrease in the number of S-phase cells observed in ponatinib-treated samples as compared with control samples (7.5% ±1% for 3μM and 7% ±1% at 5μM vs. 29% ±3% of control) (Figure 5B–5C). At the lower concentrations (1 through 5μM), ponatinib arrested merlin-deficient HSC at G_1_, indicating a cytostatic mechanism of action. However, by analyzing the live/dead populations, at a higher concentration (10μM), ponatinib became cytotoxic (Figure 5D). The increase in the number of dead cells present in the cells treated with 10μM ponatinib coincides with the decrease in phosphorylated proteins studied here.

**Figure 6:**
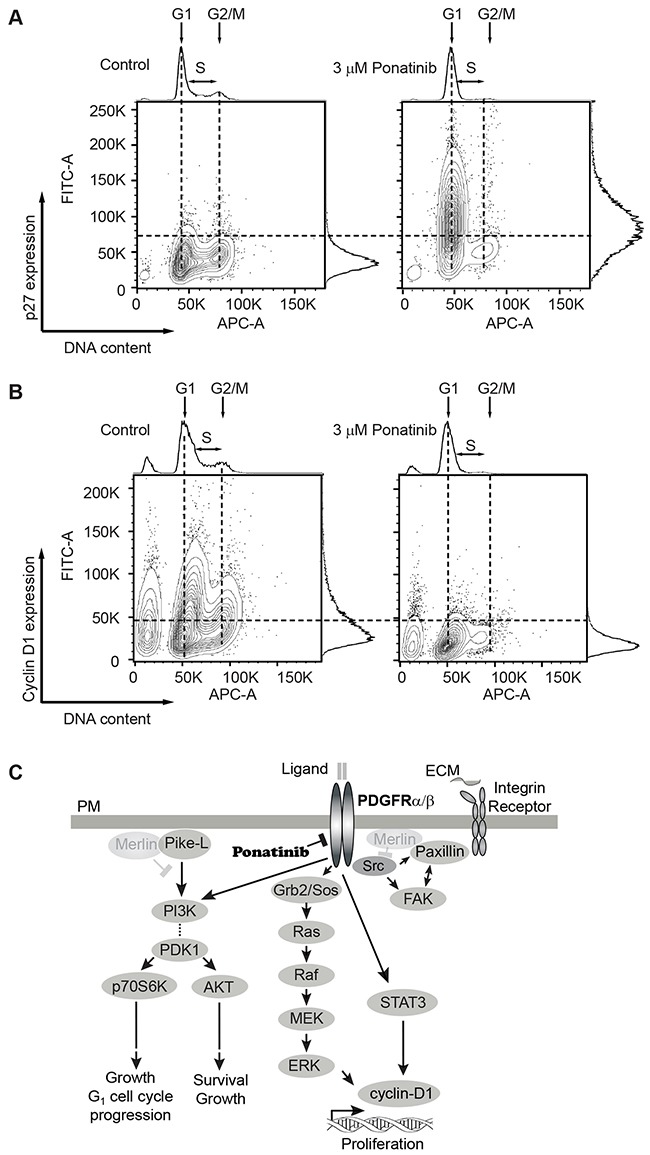
Analysis of G1 regulatory proteins during the cell-cycle in merlin-deficient HSC treated with ponatinib MD-HSC were treated with 3μM ponatinib or vehicle control for 24h. Cells were harvested, fixed, and permeabilized. DNA was stained with FxCycle, and intracellular G_1_ regulatory proteins were immunostained and analyzed by flow cytometry. **(A)** Distribution plots of cells with positive/negative p27^Kip1^ immunostain vs DNA content. Data shown are representative plots of four independent experiments. **(B)** Distribution of cells analyzed by flow cytometry with positive/negative cyclin D1 immunostain vs DNA content. Shown are representatives of four independent experiments. **(C)** Diagram of signaling pathways inhibited by ponatinib in merlin-deficient HSC. Merlin deficiency leads to activation of PDGFR, SRC and PI3K. Activation of PI3K potentiates AKT and p70S6K phosphorylation and leads to cell survival, growth and G_1_ cell cycle progression. PDGFR activity triggers ERK and STAT3 activation, leading to cyclin D1 expression and cell proliferation. SRC activation of FAK and paxillin is not modulated by ponatinib. Ponatinib decreases cell viability through downstream inhibition of AKT, ERK, and STAT3.

Lastly, we analyzed the correlation of the cell-cycle phases with levels of several cell cycle regulators. Cyclin-dependent kinase (Cdk) inhibitor p27 (p27^Kip1^) is a key negative regulator of Cdk activity in cells progressing from G_1_ toward S phase [35]. We analyzed the expression of p27^Kip1^ in conjunction with DNA content by flow cytometry. We found fewer p27^Kip1^-positive cells in G_1_ in control cells compared with a large increase in p27^Kip1^-positive cells in G_1_ in ponatinib-treated samples (Figure 6A). Similarly, we found a greater number of cyclin D1-positive cells in control samples in G_1_ in contrast to ponatinib-treated samples (Figure 6B). This result correlates with that observed from the Western blot experiment (Figure 5A). Overlay of the p27^Kip1^ and cyclin D1 plots with the cell-cycle plots clearly demonstrate that ponatinib induced G_1_ arrest with associated changes in the cyclin D1 and p27^Kip1^ levels in merlin-deficient HSC (Supplementary Figure 3AB). Our results are consistent with ponatinib inhibition of PDGFR and downstream PI3K activity leading to a G_1_ cell-cycle arrest of merlin-deficient HSC by blocking ERK- and STAT3-dependent expression of cyclin D1 (Figure 6C).

### PDGFRα/β, SRC, STAT 3 and MEK1/2 are highly phosphorylated in human vestibular schwannomas

**Figure 7:**
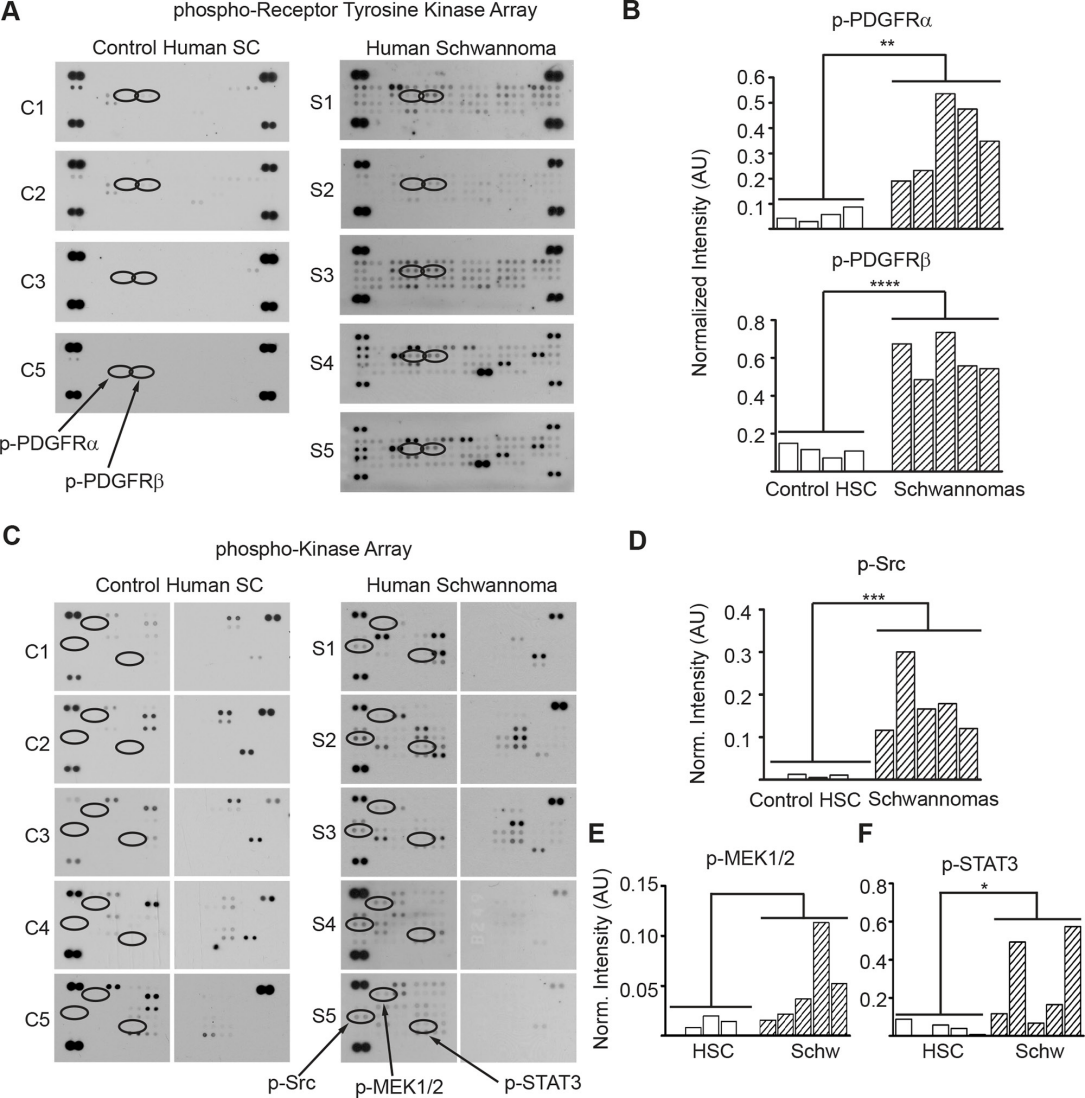
PDGFRα/β, SRC, MEK and STAT3 are overactive in human schwannomas **(A)** Phospho-RTK membrane profile of schwannomas and control cultured primary HSC. **(B)** Bar graph of the quantitation ofp-PDGFRα and p-PDGFRβ membrane dot intensity normalized to positive controls. **p<0.005; ****p<0.0001 determined using unpaired *t-*test of control HSC vs. VS populations, two-tailed. **(C)** Phospho-kinase membrane profile of schwannomas and control cultured primary HSC samples. **(D)** Bar graph of the quantitation of phospho-SRC, phospho-MEK1/2 and phospho-STAT3 membrane intensity normalized to positive controls. *p<0.05; ***p<0.001 determined using unpaired *t-*test of control HSC vs. VS populations, two-tailed.

To assess activation of PDGFRα/β and SRC in human schwannomas, we surveyed a phospho-proteome profile comparing five human vestibular schwannoma specimens with primary normal adult human Schwann cells cultured in the presence of mitogens to stimulate their proliferation. Analysis of phospho-receptor tyrosine kinase and phospho-kinases proteome profiler arrays revealed that schwannomas consistently had higher levels of phosphorylated PDGFRα, PDGFRβ, SRC, MEK and STAT3 compared with control primary HSC (Figure 7 A-D). Schwannomas exhibited averaged 6.6 times higher PDGFRα phosphorylation, 5.4 times higher PDGFRβ phosphorylation, 30 times higher SRC phosphorylation, 5.6 times higher MEK phosphorylation and 7.4 times higher STAT3 phosphorylation compared with control primary HSC (Figure 7 A-D).

## DISCUSSION

In this study, we evaluated whether ponatinib, a BCR-ABL/SRC inhibitor approved for use in leukemia, could potentially be repurposed for treatment of NF2 schwannomas. Following merlin depletion, HSC increased the levels of phosphorylated SRC and PDGFRα/β, in agreement with studies in primary human NF2 schwannoma cells [36]. Ponatinib stimulated a robust G_1_ cell cycle arrest of merlin-deficient HSC in a dose-dependent manner by inhibiting PDGFRα/β and its downstream effectors AKT, p70S6 kinase, MEK/ERK and STAT3, leading to reduced levels of cyclin D1 and increased levels of p27^Kip1^. Intriguingly, ponatinib did not reduce ABL/SRC/FAK/paxillin net phosphorylation in merlin-deficient HSC. We did not find clear evidence of ABL and SRC inhibition in merlin-deficient HSC treated with ponatinib. However, we found that ponatinib induced a dose-dependent increase in the SRC levels in the HSC. Similar results been reported for SRC inhibition with AZD0530 of Philadelphia chromosome-positive leukaemia cell lines. We speculate that this is due to a compensatory feedback mechanism, suggesting SRC kinase inhibition by ponatinib [37]. Other SRC family members, such as Fyn and Yes, did not change their protein levels in ponatinib-treated cells. In some CML cases of imatinib resistance, upregulation of SRC kinase has been implicated as a BCR-ABL-independent mechanism responsible for imatinib failure [38–40]. Therefore the increase in SRC protein levels observed ponatinib-treated HSC may be an adaptive response. It would be interesting to investigate if other SRC inhibitors, including SU6656, that have anti-proliferative activity in primary human schwannoma cells, also increase SRC levels [20, 41].

Ponatinib lacked selectivity for the merlin-deficient HSC over *NF2* wild-type HSC when cultured in the presence of serum and mitogens. This is comparable to results reported by others with the MEK1 inhibitor AZD6244 in primary human schwannoma cells and normal Schwann cells grown in serum-containing medium [21]. Notably, when cells were incubated in growth arresting medium (SCM base + N2 supplement) in the presence of 0.25 μM ponatinib for one week, merlin-deficient HSC showed a greater sensitivity to the drug than merlin-expressing HSC. To maintain fetus derived cell lines amenable to drug discovery studies, we routinely culture these embryonic Schwann cells in the presence of serum and other mitogenic supplements, which induces Schwann cells to proliferate. In contrast, both myelinating and non-myelinating Schwann cells are post-mitotic in normal nerves [42–44]. Only after nerve injury do adult Schwann cells de-differentiate and proliferate as part of the nerve repair process [21, 45–48]. Thus, ponatinib's lack of *in vitro* selectivity is not a cause for concern for *in vivo* studies and its potential as a therapeutic for NF2-associated schwannomas.

Ponatinib strongly inhibited PDGFR phosphorylation in merlin-deficient HSC. Studies with other RTK inhibitors that primarily target PDGFR and c-KIT (e.g., nilotinib, imatinib and sorafenib) showed that these RTK inhibitors exhibited strong anti-proliferative activity on primary human schwannoma and HEI-193 cells [10, 12, 41]. Merlin was shown to promote the internalization of activated PDGFRβ, a role consistent with increased expression and activation of mitogenic receptors in NF2 schwannomas [13, 14]. In Schwann cells, PDGFRα and β signaling are central to cell proliferation and survival through the Ras/Raf/MEK/ERK and PI3K/AKT signaling pathways [49–51]. In the four schwannoma samples with chronic merlin loss, the p-AKT Ser473 levels downstream of mTORC2 were reported as consistently reduced across tumor samples relative to normal arachnoid and meningioma tissues, as well as in one immortalized human SC line with acute loss of merlin achieved by an RNAi compared to the immortalized human merlin-expressing SC line [52]. The consistent decrease in mTORC2 signaling activity in schwannoma and merlin-deficient Schwann cells mimics the PDGFR activity results in schwannomas reported by others and our phospho-receptor tyrosine kinase arrays showing a high PDGFR phosphorylation levels in all tumors tested (Figure 7 and 1) [10–12]. Consistent with PDGFR inhibition, we observed a dose-dependent decrease in AKT-Thr308 phosphorylation, a PI3K-dependent phosphorylation site, and a decrease in MEK/ERK phosphorylation, similar to those reported in sorafenib-treated schwannoma cells [10, 53, 54]. Moreover, we demonstrated a dose-dependent reduction of cyclin D1 levels that correlates with STAT3 inactivation. STAT3 induces cyclin D1 transcription by binding its promoter region; and ERK1/2 has plays a critical role for induction of cyclin D1 (Figure 6C) [33, 34, 55, 56]. Decreased cyclin-D1 expression together with an increase in the p27^Kip1^ level are key to the G_1_ to S phase progression block that we observed in ponatinib-treated HSCs.

Unlike traditional dual SRC/ABL inhibitors, ponatinib is a reversible, third-generation inhibitor that binds the unphosphorylated inactive DFG-out conformation of both enzymes [57]. Ponatinib was designed to overcome resistance-inducing BCR-ABL mutations in CML and ALL treated with the first- and second-generation TK inhibitors. It is considered a pan-BCR-ABL inhibitor because it is potently active on ABL-T315I and fourteen other mutants [19]. Ponatinib shows inhibitory activity against ABL/SRC, PDGFR and other kinases, including the FGFR (fibroblast growth factor receptor), Ephrin receptors, FLT3 (FMS-like tyrosine kinase 3), VEGFR1-3 (vascular endothelial growth factor receptor 1-3), Ret (rearranged during transfection) and KIT (mast/stem cell growth factor receptor) [19]. Ponatinib therapy, similar to other TK inhibitors, is associated with severe adverse events, such as arterial thrombosis and liver toxicity, and older patients (>65 years) had higher risk of experiencing adverse effect than younger patients [58, 59]. In a large group of children and adolescents with CML treated with imatinib, assessment of long term growth revealed growth deceleration in both genders [60]. Therefore, adverse effects of long-term treatment in children should be carefully weighed.

Whereas short-term treatment with ponatinib leads to cytostasis, prolonged treatment could lead to cell death or senescence [61, 62]. These sequelae have been reported for B precursor or T cells from ALL patients treated with ponatinib; the treated cells underwent apoptosis after a G_1_ cell-cycle arrest by increasing endogenous TNF-related apoptosis-inducing ligand (TRAIL) [63]. Drugs that simply slow schwannoma growth would benefit NF2 patients enormously. If a long-term use of ponatinib is envisioned, we speculate that ponatinib may be used at lower doses in a combinatorial therapeutic approach with other compounds targeting interconnecting pathways, thereby limiting adverse effects. The different mechanisms of kinase inhibition and diverse range and selectivity of small-molecule kinase inhibitors requires that each drug be studied in NF2-relevant cell types, and weighed independently. To this extent, ponatinib treatment effectively reduced viability of merlin-deficient HSC with a robust arrest at the G_1_ phase. Ponatinib therapy may be applicable to a larger patient population than NF2, considering that merlin inactivation also occurs in sporadic schwannomas. Future *in vivo* studies addressing ponatinib alone or in combination as an effective therapy for schwannomas in NF2 mouse models are warranted.

## MATERIALS AND METHODS

### Cell cultures and human vestibular schwannomas

The merlin-deficient HSC line was generated from primary fetal HSC purchased from ScienCell (lot #7228) and authenticated by immunostaining for expression of human nuclear antigen, and SC markers, S100, PLP, O4, and Gap43, and Nestin. Cells were transduced with lentiviral particles expressing human *NF2* gene-specific shRNA (GenBank accession no. NM_000268; TRCN0000237845; TRCN0000039974; TRCN0000039975 and TRCN0000039977, Sigma-Aldrich), shRNA Scrambled, or Turbo-GFP control (Sigma-Aldrich) and then selected with 0.5 mg/ml puromycin. Wild-type and merlin-deficient HSC were cultured in CellBIND dishes (Corning) in complete Schwann cell media (SCM) from ScienCell (basal Schwann cell medium plus 5% fetal bovine serum, Schwann cell growth supplements which contains growth factors, hormones, and proteins necessary for the culture of normal human Schwann cells and 1X-penicillin/streptomycin) unless otherwise specified. Cells were used between passages 9 to 18. Merlin levels were assessed by Western blotting.

Frozen human schwannomas used in phospho-proteome studies were procured with patient informed consent at The Ohio State University College of Medicine according to Institutional Review Board regulations. Human vestibular schwannoma (VS) cells cultured to test ponatinib efficacy were isolated from dissociated fresh human tumors. Fresh VS specimens were procured with patient informed consent at University of Miami Miller School of Medicine according to Institutional Review Board regulations through the Tissue Bank Core Facility.

Normal primary human SCs, a gift from Dr. Patrick Wood (The Miami Project to Cure Paralysis, University of Miami Miller School of Medicine), were used in phospho-proteome studies, and cultured as previously described [64].

### Antibody proteome profiler arrays

Human phospho-RTK (ARY001) and phospho-kinase array (ARY003) kits were purchased from R&D systems. Human schwannoma homogenates and control HSC lysates were prepared and analyzed according to manufacturer's instructions. Arrays were visualized with chemiluminescence and were quantified with ImageJ MicroArray_Profile.jar plugin or Carestream software; mean intensity of duplicate spots was calculated.

### Antibodies and inhibitors

Rabbit antibodies against merlin (D1D8), c-ABL, phospho-AKT (Thr308; C31E5E), cyclin-D1 (92G2), FYN, p-MEK1/2 (Ser217/221), PDGFRα and β, SRC (36D10), p-SRC family (Tyr416), p-ERK1/2 (D13.14.4E), YES and mouse antibodies recognizing AKT (40D4), β-Actin (8H10D10), MEK1/2 (L38C12), ERK1/2, p27^Kip1^ (SX53G8.5) were purchased from Cell Signaling. Rabbit antibodies against p-paxillin (Tyr118) and p-FAK (Tyr577/Tyr576) were purchased from Invitrogen. Rabbit p70S6 kinase (Thr229) was purchased form ThermoFisher Scientific. Rabbit anti-p-ABL (Tyr245), and human nuclear antigen antibodies were obtained from Millipore and antibodies for myelin- proteolipid protein and GAP43 were from Abcam. The anti-S100 antibody was purchased from Dako. Mouse anti-paxillin antibody was from BD bioscience. Secondary antibodies, goat anti-rabbit IgG conjugated with DyLight 800 4X-PEG, goat anti-Mouse IgG conjugated with DyLight 680, were purchased from Cell Signaling. Ponatinib, perifosine/KRX-0401, and selumetinib/AZD6244 were purchased from SelleckChem. The STAT3 inhibitor S3I-201/NSC-74859 was purchased from MedChem Express.

### Western blot analysis

Cultured HSCs were lysed in modified RIPA buffer as previously described [65] or in 1X-SDS loading buffer plus 2.5 U/ml benzonase. 10 μg of protein or 10 μl 1X-loading buffer lysate were resolved in 4–20% polyacrylamide gels (Pierce), transferred to PVDF membranes (Immobilon-FL; Millipore), blocked with 5% BSA in TBS, and incubated with primary antibodies overnight at 4°C, and then with their corresponding fluorescence-conjugated secondary antibodies at 1:25,000-1:40,000 dilution. Image acquisition was done using LI-COR® Biosciences Odyssey® Infrared Imaging System and quantification using Odyssey Image Studio Version 3.1software and ImageJ 1.46r.

### Immunocytochemistry

Cells were grown on German glass coverslips coated with 200 μg/ml poly-L-lysine (Sigma-Aldrich). HSCs were fixed in 4% paraformaldehyde and immunostained, and stained images were acquired with a Zeiss LSM710 confocal microscope as previously described [64]. Images were processed with ZEN2011 software.

### Cell viability assay

HSC were seeded at 2,500 cells/well in 20 μl of phenol-red free SCM (SCM phenol-red free base, 5% serum, Schwann cell growth supplements containing growth factors, hormones, and proteins and 1X-penicillin/streptomycin) in 384-well plates and incubated with increasing concentrations of ponatinib in 0.1% DMSO or vehicle alone for 48h. The CellTiter-Fluor cell viability assay (Promega) was used according to manufacturer's specifications [53].

To evaluate cell viability without artificially stimulating proliferation, control and merlin-deficient HSC were cultured and assayed in growth suppressive medium (SCM base, 1X-penicillin/streptomycin –ScienCell, plus N2 supplement-Invitrogen). Cells were seeded in 24 well plates (Corning-CellBIND) at 60,000 cells/well in four replicates, after 4 days incubation at 37°C, 7% CO_2_, cells were treated with 0.25μM ponatinib or a vehicle control for one week. Viability was assessed with a crystal violet assay as previously described [64].

To evaluate cell viability of primary VS cells with *NF2* mutations, two fresh VS were obtained from the Tissue Bank Core Facility at the University of Miami Miller School of Medicine. VS tumors were cut into 1 mm pieces and dissociated in 0.5 mg/ml collagenase (~150U/ml) and dispase (2.5 mg/ml) in Dulbecco's Modified Eagle Medium (Sigma) for 1h followed by 0.25% Trypsin for 30 minutes at 37°C. Digested tissue was triturated and centrifuged at 1500 rpm at 4°C for 10 minutes. Supernatant was discarded and cells were resuspended and cultured using Schwann Cell Media (ScienCell) on culture flasks pre-treated with 0.1% poly-L-lysine (Sigma) and 25 mg/ml laminin (ThermoScientific). Cells from Passage 2 were then seeded in a 96 well plate (Costar, Corning) at 5,000 cells/well in six replicates. After 24 hours of incubation at 37°C, 5% CO2, cells were treated with ponatinib at different concentrations or 0.05% DMSO for 48h. Viability was assessed with a crystal violet assay as previously described [64].

### DNA sequencing of VS

Genomic DNA was isolated and purified using Trizol (Invitrogen) as per the manufacturer's protocol. A total of 17 pairs of primers were designed for the amplification and sequencing of the coding exons and their flanking splice sites using the Primer 3 program (http://bioinfo.ut.ee/primer3-0.4.0/). The complete coding sequences of *NF2* were amplified by PCR. DNA (~1 μg) was amplified with *NF2*-specific primer pairs in 50μL, containing 10X PCR Buffer (pH 8.5), 0.4 mM dNTP mix (Promega Corporation), 0.4 pmol/μL of each primer, and 0.0625 units of *Taq* DNA polymerase (Eppendorf AG). DNA templates were amplified using the following program: 95°C for 3 min; 35 cycles of 94°C for 50 s, 60°C for 50 s, 72°C for 60 s; and final extension of 72°C for 5 min. Direct sequencing of PCR products were performed on both strands using the ABI Prism BigDye Terminator reaction kit and ABI 3100 DNA sequencer or with Beckman Coulter 2000 XL instrument and appropriate kits.

### Cell-cycle and G_1_ proteins analysis by flow cytometry

The Click-iT EdU, FxCycle stain and Live/Dead fixable dead cell stain kits were purchased from Molecular Probes (ThermoFisher Scientific). Cells were seeded in 6-well plates and treated overnight with an inhibitor or vehicle. On the next day, 10 μM EdU was added to the cultures for 3 h, and then cells were harvested (total 24-h inhibitor incubation), stained with fixable violet live/dead stain, and permeabilized. EdU and DNA labeling was conducted according to manufacturer's instruction. Cell cycle analysis was done on gated live cells. For the cyclin D1/p27^Kip1^ expression study [66], cultures were treated with an inhibitor or vehicle for 24 h and then harvested, fixed with 4% paraformaldehyde for 10 min at 37°C, chilled for 1 min, and permeabilized for 30 min in 90% methanol. Fixed cells were transferred to a Falcon 5 ml tube through a cell strainer cap, rinsed twice with 0.5% BSA in PBS, and immunostained by incubating for 1h at room temperature with a cyclin D1 (1:400) or p27^Kip1^ (1:3,000) antibody, followed by a 30-min incubation with a goat anti-rabbit or anti-mouse-Alexa488 secondary antibody. After one wash, cells were resuspended in 1 ml of 0.5% BSA and stained for 30 min with 200nM FxCycle Far Red DNA stain supplemented with 0.1mg/ml of ribonucleaseA (Invitrogen). A BD FACS Canto-II flow cytometer (BD Biosciences) with the BD FACSDiva™ 6.1.3 software was used for data acquisition and FlowJo software was used for data analysis.

### Statistical analysis

Statistical analysis was performed using GraphPad Prism v5.0 for Windows. Ponatinib dose-response experiments were analyzed by non-linear regression (four parameters). Other experiments were analyzed by applying two-way ANOVA and Bonferroni multiple comparisons post-test, one-way ANOVA, and Dunnett's multiple comparison post-test or unpaired *t-*test, two-tailed as noted.

## SUPPLEMENTARY MATERIALS FIGURES


