# Supplementary material for: Ponatinib promotes a G1 cell-cycle arrest of merlin/NF2-deficient human schwann cells
Source: Oncotarget. 2017 Mar 6;8(19):31666–81. doi: 10.18632/oncotarget.15912 (PMC5458238; doi:10.18632/oncotarget.15912)
Supplement: Supplementary file 1 [file oncotarget-08-31666-s001.pdf]

Ponatinib promotes a G_1 cell-cycle arrest of Merlin/NF2-deficient human Schwann cells

SUPPLEMENTARY MATERIALS

SUPPLEMENTARY FIGURES

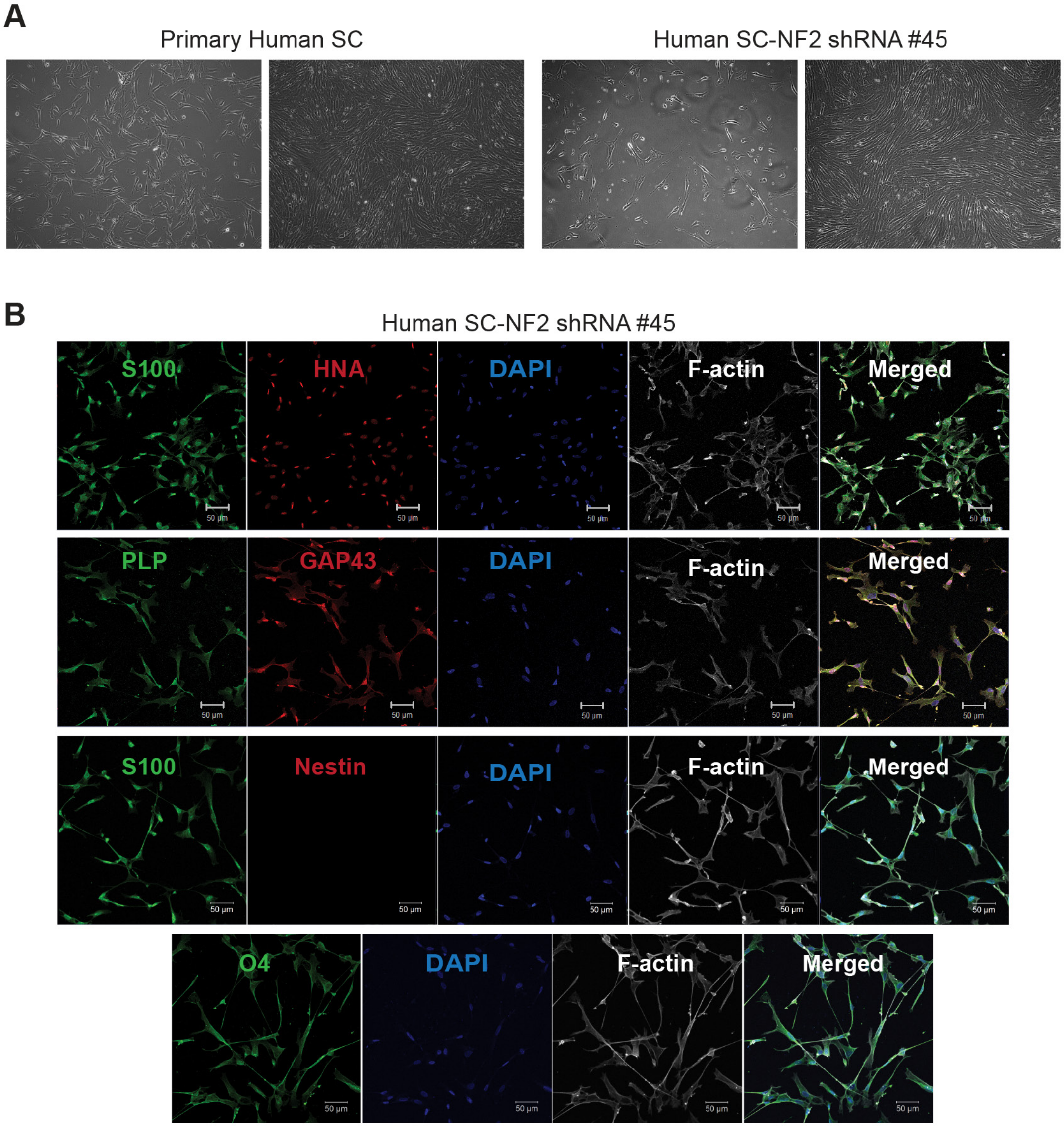

Supplementary Figure 1: Characterization of merlin-deficient human Schwann cells (MD-HSC). (A) Cell morphology: Phase contrast images of HSC lot #7228 from Science Cell before and after NF2 silencing by shRNA at low and high confluency. **(B)** Confocal Images of lot# 7228-NF2 shRNA#45-p9 (merlin-deficient HSC) displaying human and SC linage markers: HNA (human nuclear antigen), S100, GAP43, PLP (proteolipid protein), and negative nestin. DAPI (4',6-diamidino-2-phenylindole, dihydrochloride) stained nuclear DNA. Cells were seeded on poly-L-lysine (200 µg/ml) coated 12mm glass coverslips in Schwann cell medium from ScienCell at 30,000 cells/coverslip. Next day cells were fixed with 4% paraformaldehyde and subsequently stained for the expression markers indicated. Images were taken with a confocal Zeiss LSM710 microscope. Scale bar= 50µm.

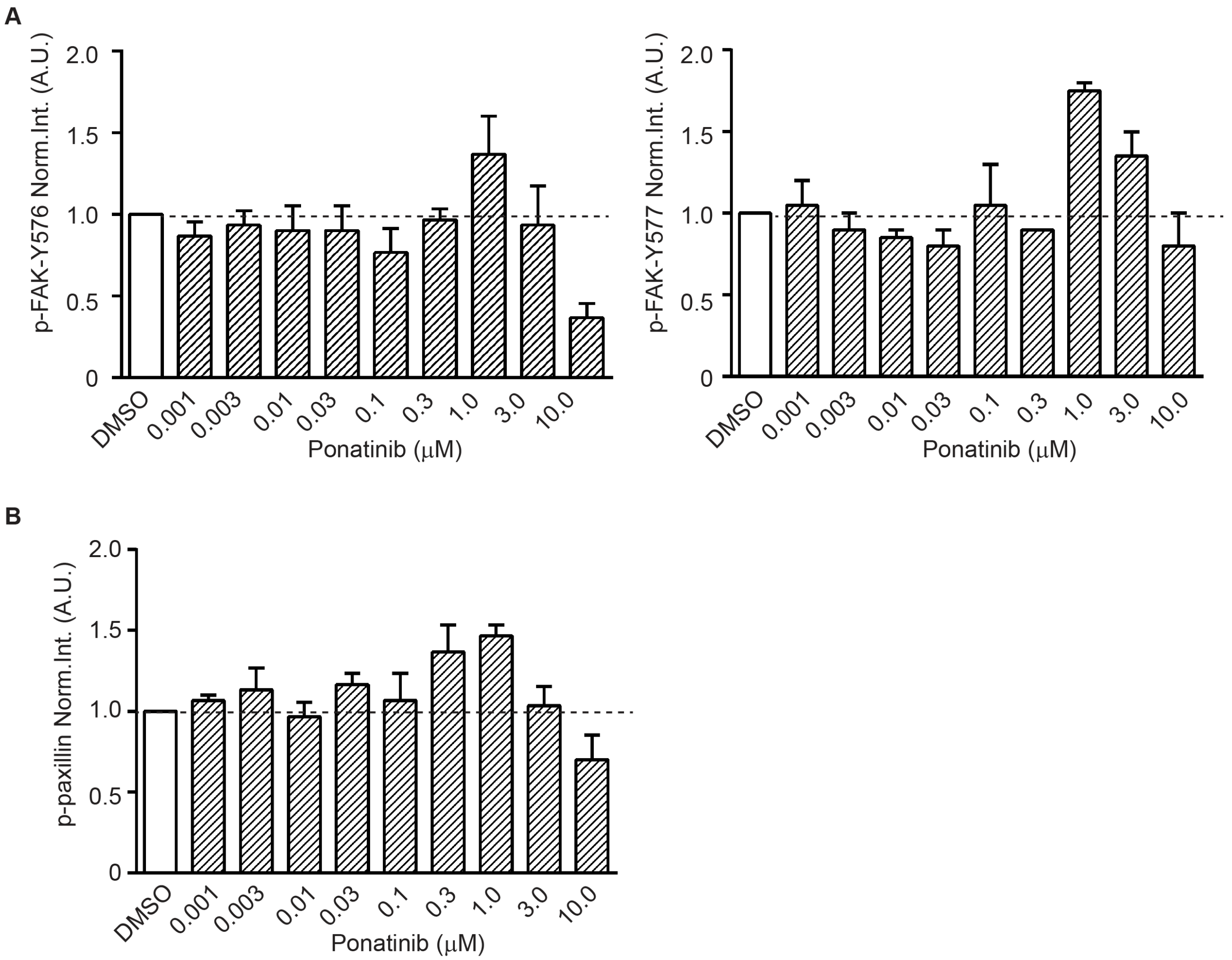

Supplementary Figure 2: Ponatinib decreased merlin-deficient HSC viability is independent of paxillin pathway inhibition. Merlin-deficient HSC#45 plated in 12-well plates were treated with increasing concentrations of ponatinib for 2h. Cells were harvested, lysed, resolved by SDS-PAGE and blotted for FAK, p-FAK-Tyr576 and -Tyr577, p-Paxillin-Tyr118, paxillin, and β-actin for loading control. Quantification of the Western blots was done by fluorescence intensity analysis and plotted as mean ± SEM (n=3). One-way analysis of variance and Dunnett's multiple comparison post-test were used for statistical analysis (no significance found). **(A)** Bar graph of the relative intensity of p-FAK-Tyr576 and -Tyr577. **(B)** Bar graph of the relative intensity of p-Paxillin-Tyr118.

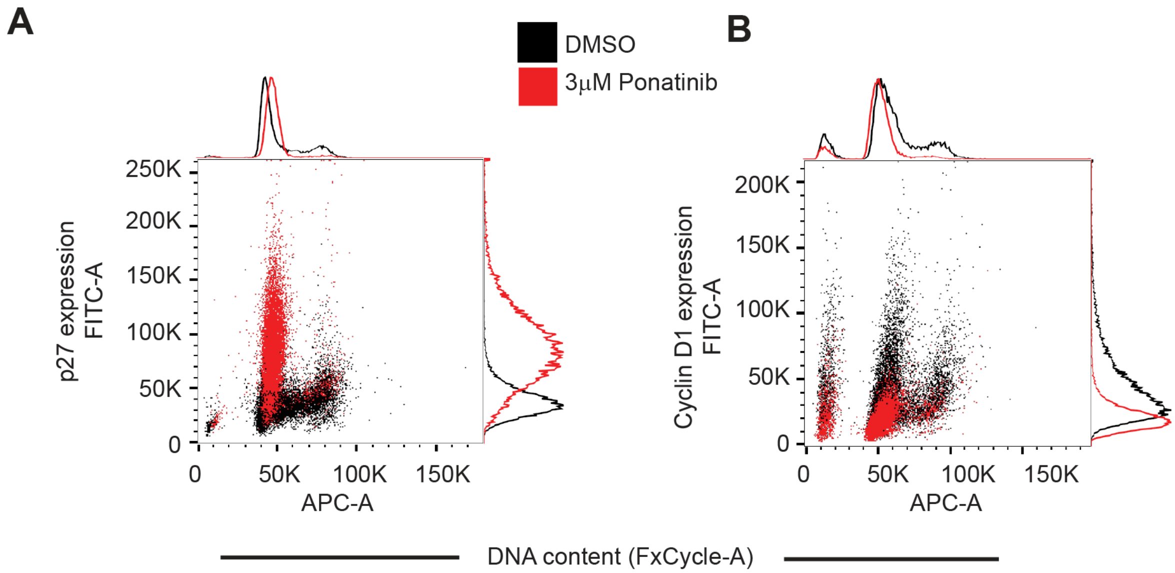

Supplementary Figure 3: Overlay of G_1 gatekeepers $p27^{kip1}$ and cyclin-D1 plots of merlin-deficient HSC treated with ponatinib or DMSO vehicle. Merlin-deficient HSC were treated ± 3μM ponatinib or vehicle control for 24h. Cells were harvested, fixed, permeabilized, DNA was stained with far red FxCycle and G1 regulatory proteins $p27^{kip1}$ and cyclin-D1 were detected by immunostaining. **(A)** Overlay of $p27^{kip1}$ plots and histograms of the FxCycle (DNA) content. **(B)** Overlay of cyclin-D1 plots and histograms of the FxCycle (DNA) content.
